# Exogenous oestrogen inhibits genital transmission of cell‐associated HIV‐1 in DMPA‐treated humanized mice

**DOI:** 10.1002/jia2.25063

**Published:** 2018-01-15

**Authors:** Nirk E Quispe Calla, Rodolfo D Vicetti Miguel, Melissa E Glick, Jesse J Kwiek, Janelle M Gabriel, Thomas L Cherpes

**Affiliations:** ^1^ Department of Comparative Medicine Stanford University School of Medicine Stanford CA USA; ^2^ The Ohio State University (OSU) College of Veterinary Medicine Columbus OH USA; ^3^ Department of Microbiology OSU College of Arts and Sciences Columbus OH USA; ^4^ OSU School of Health and Rehabilitation Sciences Columbus OH USA

**Keywords:** DMPA, genital HIV transmission, HIV prevention, humanized mice, oestrogen

## Abstract

**Introduction:**

HIV affects more women than any other life‐threatening infectious agent, and most infections are sexually transmitted. HIV must breach the female genital tract mucosal barrier to establish systemic infection, and clinical studies indicate virus more easily evades this barrier in women using depot‐medroxyprogesterone acetate (DMPA) and other injectable progestins for contraception. Identifying a potential mechanism for this association, we learned DMPA promotes susceptibility of wild‐type mice to genital herpes simplex virus type 2 (HSV‐2) infection by reducing genital tissue expression of the cell‐cell adhesion molecule desmoglein‐1 (DSG‐1) and increasing genital mucosal permeability. Conversely, DMPA‐mediated increases in genital mucosal permeability and HSV‐2 susceptibility were eliminated in mice concomitantly administered exogenous oestrogen (E). To confirm and extend these findings, herein we used humanized mice to define effects of systemic DMPA and intravaginal (ivag) E administration on susceptibility to genital infection with cell‐associated HIV‐1.

**Methods:**

Effects of DMPA or an intravaginal (ivag) E cream on engraftment of NOD‐scid‐IL‐2Rgc^null^ (NSG) mice with human peripheral blood mononuclear cells (hPBMCs) were defined with flow cytometry. Confocal microscopy was used to evaluate effects of DMPA, DMPA and E cream, or DMPA and the pharmacologically active component of the cream on vaginal tissue DSG‐1 expression and genital mucosal permeability to low molecular weight (LMW) molecules and hPBMCs. In other studies, hPBMC‐engrafted NSG mice (hPBMC‐NSG) received DMPA or DMPA and ivag E cream before genital inoculation with 10^6^ HIV‐1‐infected hPBMCs. Mice were euthanized 10 days after infection, and plasma HIV‐1 load quantified by qRT‐PCR and splenocytes used to detect HIV‐1 p24 antigen via immunohistochemistry and infectious virus via TZM‐bl luciferase assay.

**Results:**

Whereas hPBMC engraftment was unaffected by DMPA or E treatment, mice administered DMPA and E (cream or the pharmacologically active cream component) displayed greater vaginal tissue expression of DSG‐1 protein and decreased vaginal mucosal permeability to LMW molecules and hPBMCs versus DMPA‐treated mice. DMPA‐treated hPBMC‐NSG mice were also uniformly susceptible to genital transmission of cell‐associated HIV‐1, while no animal concomitantly administered DMPA and E cream acquired systemic HIV‐1 infection.

**Conclusion:**

Exogenous E administration reduces susceptibility of DMPA‐treated humanized mice to genital HIV‐1 infection.

## 1 Introduction

Women aged 15 to 24 years in sub‐Saharan Africa are especially vulnerable to HIV, and currently represent 25% of the new infections in the region 1. Reasons for this are unclear but certainly multi‐factorial, with gender‐based social, economic, political and cultural disparities possibly contributing 2. High prevalence of intergenerational sexual partnerships may play an additional role 3, and certain hormonal contraceptives appear to at least modestly increase the risk of HIV acquisition 4, 5, 6. The injectable progestins depot‐medroxyprogesterone acetate (DMPA) and norethisterone enanthate are commonly used in sub‐Saharan Africa, and women using these agents were found twice as likely to acquire HIV as women using no form of hormonal contraception 7.

Most likely, cell‐free and cell‐associated HIV‐1 are sexually transmitted, but the exact transmission frequency of each is unknown 8. Transmission of cell‐free HIV has been the more thoroughly explored, and most non‐human primate transmission studies with simian immunodeficiency virus (SIV) utilized cell‐free virus. However, in humans, HIV‐1 is more often isolated from seminal cells than seminal fluid, and cell‐associated HIV‐1 is detected in the seminal fluid of men receiving highly active antiretroviral therapy 9, 10, 11, 12, 13. Seminal fluid from healthy men contains about 10^5^ leukocytes/ml, and these numbers are markedly increased by genital infection 13, 14. These observations suggest that transmission of cell‐associated HIV‐1 can cause systemic infection 15, 16; a possibility supported in animal models. Female cats were genitally infected with cell‐associated feline immunodeficiency virus (another lentivirus with T‐cell tropism) 17, and female macaques were genitally infected with cell‐associated SIV 18. Cell‐associated HIV‐1 was also transmitted to severe combined immunodeficient (SCID) mice reconstituted with human peripheral blood leukocytes, whereas productive infection did not result from inoculating these mice with cell‐free HIV‐1 19.

It is interesting to note that multiple non‐human primate and murine models of genital HIV infection administer DMPA prior to infection to achieve uniform infectivity 20, 21. Although mechanisms responsible for this effect were not fully defined, DMPA is known to similarly enhance mouse susceptibly to genital infection with human papilloma virus and *Chlamydia* 22, 23. Offering mechanistic insight into this observation, we saw DMPA and levonorgestrel (LNG), another exogenous progestin used for long‐acting reversible contraception, increase genital mucosal permeability and susceptibility of wild‐type mice to intravaginal (ivag) infection with herpes simplex virus type 2 (HSV‐2) 24. We also found these progestin‐mediated increases in genital mucosal permeability and HSV‐2 susceptibility were abolished in mice administered DMPA and exogenous oestrogen (E) prior to infection 24. Herein, we sought to further explore these findings by comparing genital mucosal permeability and transmission of cell‐associated HIV‐1 in humanized mice administered DMPA or DMPA and E.

## 2 Methods

### 2.1 Human peripheral blood mononuclear cells

Human peripheral blood mononuclear cells (hPBMCs) were isolated by density gradient centrifugation 25, 26 from healthy donor buffy coats obtained from the Central‐Southeast Ohio Region American Red Cross. hPBMCs used to reconstitute mice in this study were determined by TZM‐bl cell assay 27, 28, 29, 30, 31, 32 to not contain infectious HIV‐1 particles.

### 2.2 Humanized mice

Prior to animal experimentation, ethical approval was obtained from The Ohio State University IACUC, and *in vivo* procedures were performed from December 2015 to September 2016 in compliance with the principles of the Guide for the Care and Use of Laboratory Animals of the Institute for Laboratory Animal Research. Six‐ to eight‐week‐old NOD‐scid‐IL‐2Rgc^null^ (NSG) female mice (n = 65) were acquired from Jackson Laboratory (Bar Harbor, ME, USA), and kept under a 12 hour to 12 hour light–dark cycle with *ad libitum* access to food and water. For NSG mouse engraftment, hPBMCs were thawed, washed and re‐suspended in PBS, and intravenously (i.v.) administered (10^7^ cells per mouse). Fourteen and 24 days after hPBMC injection, hPBMC engraftment was assessed using peripheral blood. Blood was incubated with RBC lysis buffer (eBioscience, San Diego, CA, USA), and cells stained with Live/Dead Fixable near‐IR (Invitrogen, Eugene, OR, USA) and anti‐human CD45 BV510 (H130), anti‐mouse CD45 PerCP (30‐F11) (BioLegend, San Diego, CA, USA) and anti‐human CD3 FITC (UCHT1) (BD Biosciences, San Jose, CA, USA) antibodies. Cells were fixed in Cytofix^TM^ buffer (BD Biosciences), and collected by FACSCanto II flow cytometer (BD Biosciences). Data were acquired by FACSDiva (BD Biosciences), and analysed with FlowJo software (Tree Star Inc., Ashland OR, USA). Unlike engrafted bone marrow, liver and thymus (BLT) humanized mice, no human cells are seen in lower genital tract mucosal tissues of hPBMC‐engrafted NSG (hPBMC‐NSG) mice 33. These mice (rather than BLT mice) therefore were used in the current study to explore the impact of exogenous sex steroids on vaginal mucosal integrity and genital HIV transmission, as the absence of HIV‐1 target cells in the vaginal mucosal epithelium of NSG mice minimizes the potential for confounding created by exogenous steroid‐mediated effects on human immune cell infiltration or function in the lower genital tract.

### 2.3 Exogenous steroid administration

**Figure 1:**
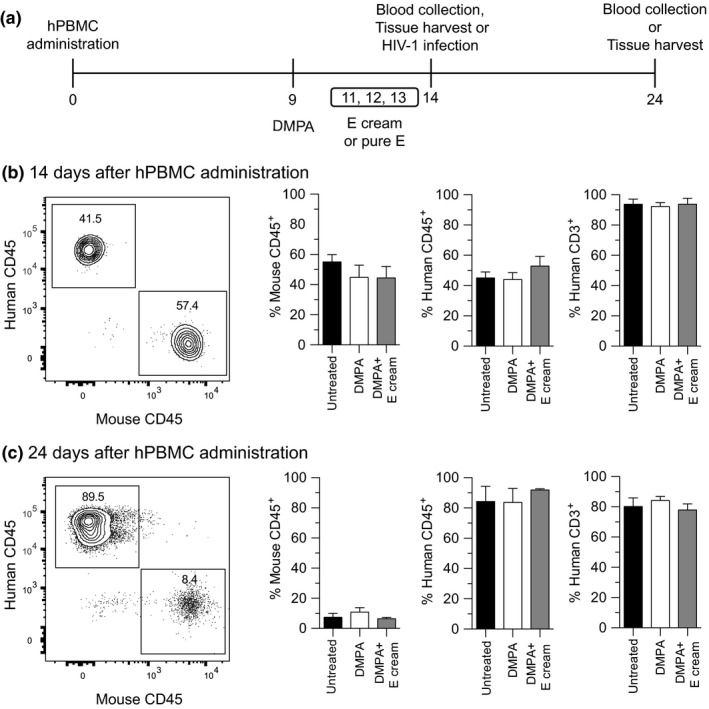
Exogenous DMPA or E did not affect hPBMC engraftment of NSG mice. (a) Schematic of the study design used to assess effects of administering DMPA and E on hPBMC engraftment, genital mucosal permeability and HIV‐1 susceptibility of NSG mice. To define effects of DMPA and E on engraftment, peripheral blood was obtained 14 days and 24 days after hPBMC administration (untreated NSG mice provided controls). (b,c) Flow cytometric studies identified no between‐group differences in the percentages of murine CD45^+^ cells and human CD45^+^ CD3^+^ cells after hPBMC administration; left panels show representative contour plots; quadrant numbers denote population percentages. Data are from 2 independent experiments with 3 animals per group (bars denote mean ± SD). Statistical analyses performed using one‐way ANOVA with Dunnett's multiple comparisons test. DMPA, depot medroxyprogesterone acetate; E, ivag oestrogen cream; hPBMC‐NSG (hPBMC‐engrafted NOD‐scid‐IL‐2Rgc^null^) mice.

hPBMC‐NSG mice were subcutaneously (s.c.) injected with 1 mg of DMPA (Depo‐Provera^®^; Pharmacia and Upjohn Co., New York, NY, USA) 5 days before mucosal permeability assays were performed or HIV‐1 transmission studies initiated (Figure 1a). This dose achieves serum levels in mice that approximate peak serum concentrations measured in women that initiate DMPA 34. As indicated, mice were ivag administered a commercially available E cream (Premarin^®^; Wyeth Pharmaceuticals Inc., a subsidiary of Pfizer Inc., Philadelphia PA, USA) or its active component (Pfizer Inc.) daily in the 3 days before permeability assays were performed or transmission studies initiated (Figure 1a). As indicated, untreated NSG mice in the oestrus stage of the oestrus cycle provided controls for experiments used to assess genital mucosal permeability and integrity.

### 2.4 HIV‐1 infection

10^6^ hPBMCs/ml were plated in RPMI‐1640 with 10% FBS, 2 mM L‐glutamine, 1 mM sodium pyruvate, non‐essential amino acids, 50 μM 2‐ME, 100 U/ml penicillin, 100 μg/ml streptomycin and 50 μg/ml gentamycin (Mediatech, Manassas, VA, USA) (hereafter termed complete media). Cells were stimulated for 48 hour in complete media containing 5 μg/ml of phytohaemagglutinin (PHA) (Sigma‐Aldrich, St. Louis, MO, USA). Cells were centrifuged, re‐suspended (2 x 10^6^ cells/ml) in complete media supplemented with 10 IU/ml recombinant human IL‐2 (rhIL‐2) (PeproTech, Rocky Hill, NJ, USA), and incubated another 5 days. hPBMCs were inoculated with 600 TCID_50_ of HIV‐1 BaL 35 for 24 hour, and re‐suspended in PBS (10^8^ cells/ml) for *in vivo* infections (portions of the HIV‐1‐infected hPBMC culture were used in a luciferase gene reporter assay to confirm HIV‐1 infectivity). For infection, hPBMC‐NSG mice were anaesthetized with xylazine and ketamine hydrochloride 36, and ivag inoculated with 10^6^ (10 μl) of HIV‐1‐infected huPBMCs. Mice were euthanized 10 days later to assess HIV‐1 infection status. Of note, mice were euthanized 24 days after hPBMC administration, while typical onset of clinical signs and mortality from graft versus host disease in NSG mice occurs no sooner than 25 days and 40 days after hPBMC engraftment respectively 37. At euthanasia, plasma was separated from blood and stored at −80°C. Approximately 2/3 of the spleen was transferred to chilled complete media, with the rest placed in buffered 4% formaldehyde for 24 hour (Thermo Scientific, Rockford, IL, USA). Splenic tissues placed in media were processed into single‐cell suspension, and cultured in complete media (10^6^ cells/ml) supplemented with rhIL‐2 (media replenished every 3 days). After 8 days, supernatants were incubated with TZM‐bl indicator cells to detect infectious HIV‐1 particles. In these assays, splenocytes from uninfected mice provided negative controls and HIV‐1 BaL diluted in complete media served as positive controls. Plasma HIV‐1 load was quantified at an OSU clinical laboratory using Abbott's real‐time PCR assay, a FDA‐approved test for HIV RNA viral load. All assays used to evaluate HIV infection status in our study were performed by investigators unaware of mouse treatment group assignment.

### 2.5 Mucosal permeability assays

To assess genital mucosal permeability to low molecular weight (LMW) molecules, sedated mice were ivag administered a 10 μl PBS solution containing 70 kDa Texas‐Red dextran and Lucifer yellow CH lithium (Invitrogen, Carlsbad, CA, USA). Mice were euthanized 45 minutes later, and fluorescent molecule penetration into vaginal tissue defined using FV1000 spectral confocal microscope system (Olympus, Center Valley, PA, USA) and ImageJ software 24, 38. To evaluate hPBMC entry into vaginal tissue, uninfected hPBMCs were activated as described above. Eight days later, cells were labelled with 5 μM of carboxyfluorescein succinimidyl ester (CellTrace CFSE; Life Technologies, Carlsbad, CA, USA), re‐suspended in PBS (10^8^ cells/ml), and mice ivag inoculated with 10 μl of this suspension. After 15 hour, mice were euthanized, vaginas excised, and tissues fixed in formaldehyde, agarose‐embedded and DAPI stained. The fluorescent signal of CSFE‐labelled hPBMCs was used to assess depth of leukocyte infiltration into vaginal submucosal tissue using confocal microscopy and ImageJ software. Confocal images were acquired by sequential scanning to prevent fluorescence crossover.

### 2.6 DSG‐1 protein and HIV‐1 p24 antigen

To assess desmoglein‐1 (DSG‐1) protein expression, vaginal tissue excised from euthanized mice was fixed in buffered formaldehyde. DSG‐1 expression was quantified as described earlier 24. Using methods described previously, HIV‐1 p24 protein expression was defined in splenic tissue sections after de‐paraffinization and antigen retrieval, overnight blocking with 5% BSA, and incubation with rabbit monoclonal anti‐CD45 (EP322Y) and goat polyclonal anti‐HIV p24 (ab53841) (Abcam, Cambridge, MA, USA) 24. Samples were processed for confocal microscopy analysis as detailed above.

### 2.7 Statistical considerations

All statistical analyses were performed using Prism 6 software (GraphPad, La Jolla, CA, USA), with normality assessed using evaluation of the residuals. For comparisons between 2 groups, unpaired Student's *t*‐tests were used. For comparisons between multiple groups, one‐way ANOVA with Dunnett's post hoc test or Kruskal–Wallis test with Dunn's post hoc test were used (depending on data distribution) (*p* ≤ 0.05 were deemed statistically significant).

## 3 Results

### 3.1 DMPA and E administration did not affect hPBMC engraftment of NSG mice

While the primary objective of this study was to define the effects of treatment with DMPA and E on susceptibility of hPBMC‐NSG mice to genital HIV‐1 infection, we first needed to determine if these compounds altered hPBMC engraftment. Using peripheral blood from untreated controls, DMPA‐treated and DMPA‐ and E cream‐treated NSG mice 14 days and 24 days after hPBMC administration (Figure 1a), our flow cytometric analyses identified more robust engraftment of human CD45^+^ CD3^+^ cells at the latter time point. However, comparing untreated controls and mice administered systemic DMPA or DMPA and ivag E, neither time point was associated with statistically significant differences hPBMC engraftment (Figure 1b,c).

### 3.2 Exogenous E prevented DMPA‐mediated loss of genital mucosal barrier function

**Figure 2:**
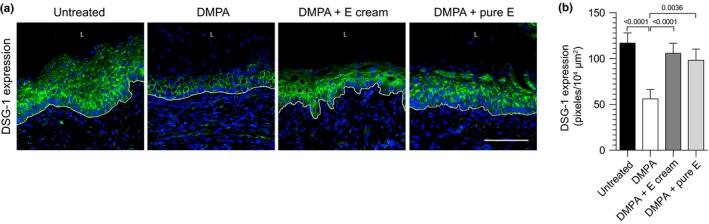
DMPA‐mediated reduction in vaginal DSG‐1 protein expression was abrogated in hPBMC‐NSG mice administered DMPA and E. hPBMC‐NSG mice were untreated or treated with DMPA, DMPA and ivag E cream, or DMPA and ivag pure E. As detailed in Methods, vaginal tissue was collected from euthanized mice to quantify DSG‐1 protein expression. (a) Representative confocal microscopic images of vaginal DSG‐1 protein expression; L (vaginal lumen); DAPI (Blue); DSG‐1 (green); white line delimits the vaginal mucosal epithelium; scale bar denotes 100 μm. (b) Quantification of DSG‐1 protein expression showed significantly reduced levels in hPBMC‐NSG mice administered DMPA alone. Data from 2 independent experiments with 3 animals per group (bars denote mean ± SD). Statistical analyses performed using one‐way ANOVA with Dunnett's multiple comparisons test. hPBMC‐NSG (hPBMC‐engrafted NOD‐scid‐IL‐2Rgc^null^) mice; DMPA, depot medroxyprogesterone acetate; E, vaginal oestrogen cream; pure E, pharmacologically active component of E cream; DSG‐1, desmoglein‐1.

Because DMPA and E did not affect hPBMC engraftment of NSG mice, we explored the effects of these compounds on genital mucosal barrier function. We first defined effects of DMPA and E on vaginal expression of DSG‐1, a cell‐cell adhesion molecule needed to maintain barrier function in cutaneous and intestinal epithelium 39, 40. In earlier work with wild‐type mice, we established that systemic DMPA treatment significantly reduced vaginal tissue expression of DSG‐1 without affecting levels of other cell‐cell adhesion molecules expressed in genital mucosa, including tight junction protein 1, claudin‐1 and occludin 24. In the current study, we measured vaginal tissue expression of DSG‐1 protein in hPBMC‐NSG mice that were: oestrus stage and untreated with exogenous sex steroids; DMPA‐treated; or administered DMPA and ivag E cream. To exclude the possibility that physical properties of the E cream produced any observed effect, other hPBMC‐NSG mice received DMPA injection and ivag administration of the pharmacologically active component of E cream (i.e. pure E). These studies revealed comparable DSG‐1 protein expression in untreated control mice and hPBMC‐NSG mice administered DMPA and oestrogen (E cream or pure E), but significantly reduced DSG‐1 expression in vaginal tissue of mice treated with DMPA alone (Figure 2).

**Figure 3:**
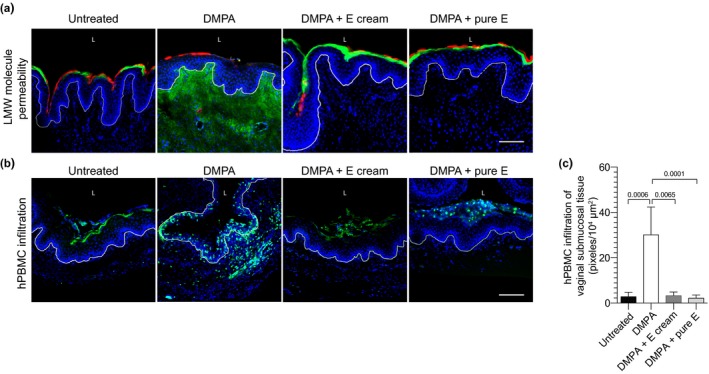
Combined treatment with DMPA and exogenous E obviated DMPA‐mediated increases in vaginal mucosal permeability. hPBMC‐NSG mice remained untreated or were treated as described in Figure 2. As detailed in Methods, vaginal tissue was excised from euthanized mice to assess permeability to LMW molecules or CFSE‐labelled hPBMCs. (a, b) Representative images illustrate increased permeability to LMW molecules and activated human leukocytes in hPBMC‐NSG mice administered DMPA vs. untreated controls or animals administered DMPA and ivag E; scale bars denote 100 μm. (a) L (vaginal lumen); DAPI (blue) Lucifer Yellow (green); 70 kDa Texas‐Red dextran (red), and (b) L (vaginal lumen); DAPI (blue); CFSE‐labelled hPBMCs (green); white line delimits vaginal mucosal epithelium. (c) Quantifying depth of hPBMC infiltration into vaginal submucosal tissue identified significantly deeper infiltration in mice administered DMPA alone. Displayed data from 2 independent experiments with 3 animals per group (bars denote mean ± SD). Statistical analyses performed using one‐way ANOVA with Dunnett's multiple comparisons test. hPBMC‐NSG (hPBMC‐engrafted NOD‐scid‐IL‐2Rgc^null^) mice; DMPA, depot medroxyprogesterone acetate; CFSE, carboxyfluorescein succinimidyl ester; E, oestrogen cream; pure E, pharmacologically active component of the E cream.

In follow‐up studies, we used identically treated groups of mice to delineate the effects of systemic DMPA and ivag E administration on vaginal mucosal permeability to LMW molecules and activated human leukocytes. Compared to DMPA‐treated hPBMC‐NSG mice, these studies identified reduced mucosal penetration of fluorescent LMW molecules and leukocytes in untreated mice and hPBMC‐NSG mice administered DMPA and ivag E or DMPA and pure E (Figure 3). Because we identified similar vaginal tissue expression of DSG‐1 protein and comparably reduced vaginal tissue penetration of LMW molecules and activated human leukocytes among hPBMC‐NSG mice treated with DMPA and E cream or pure E (vs. mice treated with DMPA alone), these studies also resolved E itself was responsible for strengthening genital mucosal barrier function.

### 3.3 Exogenous E abrogated susceptibility of DMPA‐treated humanized mice to genital transmission of cell‐associated HIV

**Figure 4:**
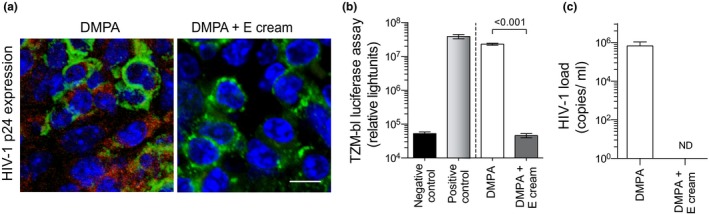
Exogenous E reversed susceptibility of DMPA‐treated humanized mice to genital transmission of cell‐associated HIV‐1. hPBMC‐NSG mice were treated with DMPA or DMPA and ivag E cream and genitally inoculated with cell‐associated HIV‐1 (as described in Methods and depicted in Figure 1a). Ten days after genital inoculation, mice were euthanized to assess HIV‐1 infection status. (a) Representative images of immunostaining for HIV‐1 p24 antigen in the spleens of euthanized mice show this viral protein detected only in mice treated with DMPA alone; DAPI (blue); anti‐human CD45 (green); anti‐HIV‐1 p24 antigen (red); scale bar denotes 20 μm. (b) TZM‐bl luciferase assay identified infectious HIV‐1 particles only in spleens from DMPA‐treated mice (splenocytes from uninfected mice and HIV‐1 BaL diluted in media provided negative and positive controls respectively). (c) A qRT‐PCR assay detected HIV‐1 virus only in the serum of hPBMC‐NSG mice administered DMPA alone (ND denotes no virus was detected). Data displayed are from 2 independent experiments with 5 animals per group (bars denote mean ± SD). Statistical analyses were performed using the unpaired Student's *t*‐test. DMPA, depot medroxyprogesterone acetate; E, vaginal oestrogen cream; hPBMC‐NSG (NOD‐scid‐IL‐2Rgc^null^) mice.

Because ivag E or pure E restored DMPA‐mediated loss of mucosal barrier function, we hypothesized exogenous E eliminates the susceptibility of hPBMC‐NSG mice to genital transmission of cell‐associated HIV‐1. To explore this hypothesis, hPBMC‐NSG mice were systemically treated with DMPA or DMPA and ivag E cream (Figure 1a). All mice were genitally inoculated with 10^6^ HIV‐1‐infected hPBMCs, and euthanized 10 days later to assess HIV‐1 infection status. Immunohistochemical tests for HIV‐1 p24 antigen detected this protein only in the spleens of DMPA‐treated hPBMC‐NSG mice (Figure 4a). Using a TZM‐bl luciferase assay to qualitatively detect the presence of infectious HIV‐1 particles, we detected significantly increased signal in the spleens of DMPA‐treated mice versus mice treated with DMPA and the ivag E cream (Figure 4b). These results indicated that systemic HIV‐1 infection was prevented by combined DMPA and ivag E treatment. Offering further support for this conclusion, HIV‐1 virus copies were detected by qRT‐PCR assay in the plasma of hPBMC‐NSG mice administered DMPA, but not in the plasma of hPBMC‐NSG mice treated with DMPA and E (Figure 4c).

## 4 Discussion

Male‐to‐female HIV transmission rates approximate 0.12% per sex act 41, implying the virus must overcome host defences in the female genital tract to establish systemic infection. As examples, virus particles that avoid entrapment in epithelial surface mucus must breach the mucosal epithelium to interact with submucosal tissue target cells 42, 43. While pathogen‐induced ulcers and coital abrasions may help HIV‐1 evade genital mucosal barriers 44, 45, current findings suggest DMPA‐mediated increases in genital mucosal permeability also promote virus transmission. Compared to hPBMC‐NSG mice administered DMPA and E, vaginal tissue of mice treated with DMPA displayed reduced expression of DSG‐1 protein and impaired mucosal barrier function, and DMPA‐treated hPBMC‐NSG mice were highly susceptible to genital transmission of cell‐associated HIV‐1. These findings thus corroborate results in which DMPA‐ and LNG‐treated wild‐type mice displayed increased genital mucosal permeability and susceptibility to cell‐free HSV‐2 infection 24. Current findings also corroborate clinical data in which ectocervical DSG‐1 expression and mucosal permeability were analogously altered in women initiating use of DMPA or a LNG‐releasing intra‐uterine system 24, 46.

While our results may identify an underlying mechanism by which exogenous progestins enhance HIV susceptibility, they cannot exclude contributions from other mechanisms. Several clinical studies saw greater inflammation in genital tissue after women initiated DMPA 24, 47, 48, and it is possible that DMPA‐mediated increases in genital inflammation can enhance HIV susceptibility. On the other hand, mouse model findings reveal that DMPA‐mediated increases in genital inflammation occurred downstrearm of DMPA‐mediated increases in genital mucosal permeability that facilitated tissue invasion by endogenous microbiota 24. Moreover, while DMPA makes hPBMC‐NSG mice uniformly susceptibly to genital HIV‐1 transmission (Figure 4), genital tract submucosal tissue in these mice does not contain human immune cells 33, 49. This implies that the enhanced HIV susceptibility of DMPA‐treated hPBMC‐NSG mice was not created by DMPA‐mediated inflammatory responses that increased the frequency of HIV target cells. While current results offer novel indication that DMPA‐mediated impairment of genital mucosal barrier function promotes HIV transmission, actual contribution of this effect to similarly enhancing HIV susceptibility of women using DMPA awaits further investigation.

On the other hand, DMPA‐mediated enhanced susceptibility of hPBMC‐NSG mice to cell‐associated HIV‐1 infection is congruent with previous reports that DMPA increased susceptibility of hPBMC‐SCID mice and non‐human primates to atraumatic genital inoculation with cell‐associated HIV‐1 and SIV, respectively 19, 50. Our current findings provide important extension of these results, showing that treatment of mice with ivag E improves genital mucosal barrier function and protects DMPA‐treated mice from cell‐associated HIV‐1 acquisition. While non‐human primate studies identified that exogenous E reduced susceptibility to genital SIV infection 51, 52, our findings appear to be the first to demonstrate that exogenous E abrogates HIV‐1 susceptibility in DMPA‐treated animals. Current results further establish the enhanced barrier protection was a direct effect of E, as ivag administration of an E cream (Premarin^®^) and pure Premarin^®^ substance similarly increased vaginal expression of DSG‐1 protein and reduced genital mucosal permeability to LMW molecules and hPBMCs. These findings imply the ability of ivag E cream to protect DMPA‐treated hPBMC‐NSG mice from cell‐associated HIV‐1 infection was not an artefact of cream impeding access of HIV‐infected hPBMCs to the genital epithelial surface. Considered in combination with prior results 24, the capability of exogenous E to protect progestin‐treated mice from cell‐free and cell‐associated genital virus infection implies that exogenous E and progestin may be components of a hormonal contraceptive platform that is less compromising of genital mucosal barrier function than those that deliver progestin unopposed. However, as there are important differences in the genital tract of mice and women, including the fact that rodent vaginal epithelium is keratinized and human vaginal epithelium is not 53, it will be critical to define efficacy of contraceptive platforms releasing exogenous E and progestin in highly relevant clinical models, including non‐human primates.

## 5 Conclusion

Our studies show that DMPA weakens genital mucosal barrier function. As barrier function is such a fundamental anti‐virus host defence, any factor that weakens this protection may represent an important HIV risk factor. Our studies also provide new biological plausibility for the putative link between DMPA and HIV susceptibility, and suggest that use of unopposed progestins for hormonal contraception may impede efforts to curb the HIV pandemic. On the other hand, we identified that exogenous E reverses DMPA‐mediated increases in mucosal permeability and HIV susceptibility. Based on the ability of exogenous E to eliminate susceptibly of DMPA‐treated humanized mice to genital HIV‐1 infection, combined use of exogenous progestin and E may provide basis for hormonal contraceptive approaches among women at higher risk for HIV acquisition, particularly in more resource‐limited settings. However, defining the safety and efficacy of contraceptive platforms that use exogenous E to boost genital mucosal barrier function requires new clinical study and further exploration of clinically relevant animal models.

## Competing interests

Authors have no competing interests to declare.

## Authors’ contributions

Study design and data acquisition, analysis and interpretation performed by N.E.Q.C., R.D.V.M., J.J.K, and T.L.C.; M.E.G. and J.M.G. performed and analysed some experiments; manuscript originally drafted by N.E.Q.C, with all authors contributing to final version.

## Funding

Funding was provided by the Eunice Kennedy Shriver National Institute of Child Health and Human Development (grant R01HD072663), OSU College of Medicine, and Stanford University School of Medicine.
